# Characterization of the Increase in Narcolepsy following the 2009 H1N1 Pandemic in Sweden

**DOI:** 10.3390/jcm13030652

**Published:** 2024-01-23

**Authors:** Helena Gauffin, Inger Boström, Shala Ghaderi Berntsson, Anna Kristoffersson, Mats Fredrikson, Anne-Marie Landtblom

**Affiliations:** 1 Department of Biomedical and Clinical Sciences, Faculty of Medicine and Health, Linköping University, 58185 Linköping, Sweden; helena.gauffin@regionostergotland.se (H.G.); bostrom.i@live.se (I.B.); mats.fredrikson@liu.se (M.F.); anne-marie.landtblom@neuro.uu.se (A.-M.L.); 2 Department of Medical Sciences, Neurology, Uppsala University, 75185 Uppsala, Sweden; anna.kristoffersson@neuro.uu.se

**Keywords:** narcolepsy, 2009 H1N1 pandemic, vaccination campaign, prevalence

## Abstract

(1) Background: In the context of the H1N1 pandemic and the Pandemrix vaccination campaign, an increased number of narcolepsy cases were noted in several countries. In Sweden, this phenomenon was attributed to the effect of the Pandemrix vaccination in the first place. Studies from China indicated that narcolepsy could occur as a consequence of the H1N1 infection itself. We performed an analysis of the increase, with a specific interest in age and sex distribution. We also aimed to validate the origin of the excess cases, post hoc. (2) Methods: Data for narcolepsy patients (ICD code G 47.4, both type 1 and type 2) distributed by sex and age at 5-year intervals, annually between 2005 and 2017, were retrieved from the National Patient Register. Information on the total population was collected from the Swedish Population Register. (3) Results: The number of narcolepsy cases increased markedly from 2009 to 2014 compared to the period before 2009. A particular increase in 2011 among children and teenagers was observed. The sex ratio did not change significantly during the study period. (4) Conclusions: Our results support an association between the increased prevalence of narcolepsy cases and Pandemrix vaccination, but the effect of the virus itself cannot be ruled out as a contributing factor.

## 1. Introduction

Narcolepsy is a chronic neurological disorder affecting rapid eye movement (REM) sleep. It is characterized by extended daytime sleepiness, fragmented sleep, nightly hallucinations, and sleep paralysis [1,2]. A key symptom is cataplexy, a loss of muscle tone elicited by emotions, especially humor, and jocularity [3]. Cognition is frequently affected and the condition has severe socioeconomic consequences [4,5,6,7]. Narcolepsy is categorized into two types. Narcolepsy with associated cataplexy is classified as narcolepsy type 1; narcolepsy without cataplexy is type 2 [8]. Patients with narcolepsy type 1 have decreased hypocretin neurons in the lateral hypothalamus, providing a pathogenetic explanation, as hypocretin plays an important role in the human sleep and metabolic system [9,10].

Publications on the prevalence of narcolepsy report diverse rates and are difficult to compare. A global review reported a mean prevalence of 30/100,000, with ranges between 4 and 56/100,000 inhabitants and even up to 160/100,000 in Japanese children [11,12]. This large variation is explained by the disparity between populations of different ethnicities, age groups, and methodologies. In Sweden, a recent study mentioned an unexpectedly low crude prevalence of 21.7/per 100,000 inhabitants in 2016, restricted to healthcare-associated cases, retrieved from the National Patient Register (NPR) and pharmaceutical registries [6]. Studies on the general incidence of narcolepsy have shown variable numbers as well, with peaks in childhood/adolescence but also later in life [13,14]. A previous review concerning Sweden estimated higher numbers, i.e., an incidence rate of 0.3–0.6/100,000/year, suggesting an estimated number of 30–60 cases/year [15]. A study by Wijnans et al. described the morbidity of narcolepsy in Europe before the influenza A (H1N1) pandemic and shortly after, with a pooled incidence in Sweden of 1/100,000 [16].

Sex ratio has shown varying distributions, and a large international study that characterized clinical presentations of narcolepsy in different ethnicities showed no difference in sex ratio across ethnicities [17]. A US healthcare claims database, from 2008 to 2010, showed a 50% greater prevalence and incidence among women compared to men across most age groups [18]. Another population-based study of narcolepsy incidence from 2004 to 2013 in the US also estimated a higher rate among women [19]. On the other hand, a Chinese study analyzing 162 patients with narcolepsy found that narcolepsy occurred 1.73-times more frequently in men than women [20]. A recent study by the National Patient Register found a sex ratio of 1.3 (women/men), i.e., a slight dominance of women in Sweden [6].

The causes of narcolepsy are multifactorial, with genetic susceptibility through a strong association with the human leucocyte receptor (HLA) DR2 [21,22] and several environmental factors of importance [23].

Infections such as streptococcal infections and influenza A have been reported to precede the debut of narcolepsy [24,25]. Subsequently, there has been strong evidence for narcolepsy being an autoimmune disorder, even though no useful auto-antibodies have been identified in clinical settings [26,27].

In the context of the H1N1 pandemic and the Pandemrix vaccination campaign in 2009–2010, an increased number of narcolepsy cases were noted in several countries [28]. Children affected by narcolepsy after the vaccination were reported to have a more sudden onset of the disease compared to previously known cases. Sweden had a high vaccination rate of around 60% of the population, and the subsequent increase in narcolepsy was eventually interpreted as an effect of the extensive Pandemrix vaccinations [29,30,31,32]. Notably, in China, where no vaccinations were performed, studies also indicated an increase in seasonal narcolepsy incidence, most likely due to the H1N1 infection [33]. Furthermore, in Denmark, a country with a low vaccination rate, an increasing number of patients with narcolepsy were identified during the period after the H1N1 infection [34]. Consequently, the correlation between the swine flu virus H1N1 infection and/or Pandemrix vaccination and the growing incidence rate of narcolepsy is of interest in both cases.

The background to this article was neurologists’ recent clinical impression that narcolepsy patients in the adult neurology outpatient clinics were getting steadily younger. This seemed to be supported by regional data in the Swedish Narcolepsy Registry (NARKREG), a sub-registry of the national Neuroregistry launched in 2012 that collects narcolepsy cases at specialized care clinics.

The purpose of NARKREG is to support good and equal quality of care for narcolepsy patients in our country by monitoring clinical variables, treatment, and outcome measures (www.neuroreg.se, (accessed on 18 January 2024)). Here, statistical instruments along with digital visualization platforms are used to demonstrate the quality of narcolepsy health care, including treatments and outcome measures that can be compared between different regions, with the specific aim of developing equal health care.

Our aim was to analyze the increase in narcolepsy cases in Sweden after the H1N1 pandemic and Pandemrix vaccination, with a focus on age and sex distribution by using the National Patient Register (NPR). We aimed to post hoc validate the origin of the excess cases.

## 2. Materials and Methods

Data on narcolepsy patients were extracted from the NPR (https://socialstyrelsen.se accessed on 22 May 2019). The dataset included the number of individuals with narcolepsy type 1 and type 2 (ICD code G47.4) annually from 2005 to 2017, categorized by sex and age groups spanning five years, and this period covered four years before and eight years after the H1N1 Pandemic. The application of the ICD code has been the same since 1998. Data were grouped into five age groups (0–19, 20–39, 40–59, 60–79, and ≥80). The prevalences in these age groups were calculated by dividing the number of cases of narcolepsy by the population for each age group.

Since 1968, the NPR has collected data on all inpatient visits to the hospital in a mandatory manner, and after 2001 this was expanded to include all outpatient specialist care visits. Accordingly, all patients followed by specialists in hospitals are included. However, primary health care is not included in this register.

The Swedish Total Population Register (TPR) collects and provides information on all persons born in 1932 or later in Sweden. For the prevalence calculation, the information obtained on the number of individuals was divided into sex and age groups annually from 2005 to 2017 in Sweden (https://www.scb.se (accessed on 15 March 2021)).

Stata v17 (StataCorp LLC, College Station, TX, USA) was used for the statistical analysis of the trend in the prevalence over the years, using a score test to determine the trend of odds.

In a post hoc analysis, the excess cases were calculated using a stipulated incidence rate of 0.7/100,000/year, corresponding to a crude number of about 70 cases/year in Sweden; a natural incidence calculated from data published by Wijnans et al. [16]. Their study included the year after the swine flu and the Pandemrix vaccination. We therefore extracted the number of cases forming the incidence before September 2009 from the material published in their article [16]. We considered that the long observation time from 2000 to September 2008 was sufficient for calculating a mean incidence of 0.7/100,000 in our country before the pandemic.

The study was approved by the Institutional Review Board in Linköping (Dnr 2014/129–31, approved on 26 March 2014), (Dnr 2020–02594, approved on 16 July 2020).

## 3. Results

Calculations from the Swedish narcolepsy registry (NARKREG) in the period between 2005 and 2017 revealed 950 patients with both a low mean age (37.4 years) and a low median age (31 years) of narcolepsy patients treated in specialist care, supporting the clinical impression of a decrease in age.

The data extracted from the NPR showed that the number of narcolepsy cases in the study cohort in 2005 consisted of 637 individuals (375 women; 262 men), yielding a crude prevalence of 7.04 per 100,000 inhabitants (women 8.2; men 5.8). The number of narcolepsy patients increased significantly between 2009 and 2014 compared to the period before 2009. The most significant increase was seen in 2011, with a total number of 1341 patients (763 women; 578 men), corresponding to a prevalence of 14.1 per 100,000 inhabitants (women 16.0; men 12.2). In 2017, the total number of narcolepsy patients was 2044 (1169 women; 875 men), with a total prevalence of 20.2/100,000 (women 23.2; men 17.2) (Table 1).

The prevalence was higher among women than men. The sex ratio in the total cohort 2017 was 1.3 (w/m), *p* < 001. Notably, the sex ratio remained unchanged during the study period and was approximately the same after the H1N1 pandemic and the Pandemrix vaccination campaign (Table 1).

**Figure 1:**
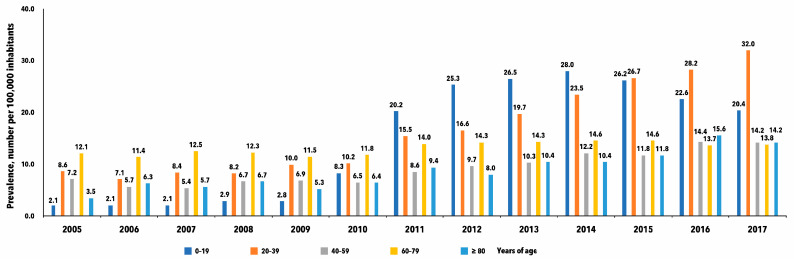
Annual study cohort extracted from the National Patient Register (NPR), showing the prevalence of narcolepsy for different age groups from 2005 to 2017 in Sweden.

The overall prevalence of narcolepsy cases increased significantly between 2005 and 2017 (*p* < 0.01) Table 1. An increasing prevalence was particularly noted in the younger age groups (0–19 years), starting in 2010, with a prevalence of 8.3/100,000 inhabitants and an annual increase to 28.0 in 2014. In the age group 20–39 years, the prevalence was 15.5/100,000 inhabitants in 2011, with a further increase to 32/100,000 inhabitants in 2017, (*p* < 0.01), Figure 1 and Supplementary Table S1.

## 4. Discussion

The number of patients diagnosed with narcolepsy in Sweden increased significantly after the H1N1 pandemic and the Pandemrix vaccination in 2009–2010, which is confirmed by our data. This was especially evident in children and teenagers, as visualized in Figure 1 and demonstrated by previous studies and other methods, including our report on children in Östergötland county in Sweden, which showed an odds ratio as high as 17.7 (95% CI: 2.7–147.5; *p* = 0.0036) [29]. In Sweden, however, only 450 persons with narcolepsy have received economic compensation for these side effects of the Pandemrix vaccination. In addition, our clinical impression was that vaccinated narcolepsy cases tended to dominate in the Swedish narcolepsy polyclinic, with or without approved compensation. This prompted us to analyze the increase more thoroughly, focusing on age and sex.

### 4.1. The National Patient Register (NPR)

The observed increase in the number of narcolepsy patients started from an unexpectedly low level, about 800 (2005–2009), which may be explained by the register’s recent inclusion of specialist open care. The NPR register did not include patients from open visits in neurology until 2001; i.e., only four years before the first data in the present study. Importantly, patients with a previous narcolepsy diagnosis who were neither actively seeking care nor using pharmacotherapy were excluded. Patients attending primary health care were also left out, since these visits are not registered in the NPR. However, we believe that this number should be small, since general practitioners usually do not diagnose or treat patients with narcolepsy and typically do not possess the required license for prescribing the drugs that are used to treat narcolepsy. The rather stable baseline prevalence before the pandemic in the period 2009–2010 incentivized us to use the NPR data for comparison when analyzing the increase in narcolepsy patients.

### 4.2. Persisting Increase in the Prevalence of Narcolepsy

The increase in the prevalence of narcolepsy was most prominent between 2009 and 2014, but even later in 2017 the prevalence of narcolepsy in Sweden was slowly increasing and had not yet reached a plateau. Notably, to receive economic compensation from Swedish Pharmaceutical Insurance and the Swedish authorities, the onset of narcolepsy must have been documented within two years after the patient’s Pandemrix vaccination, i.e., around 2012. However, the prevalence has continued to increase long after that time. Delayed diagnosis, a well-known phenomenon, has contributed to this trend. One example is that hypersomnia in teenagers is often mistaken as a natural phenomenon and a narcolepsy diagnosis is therefore not considered [35]. This is a well-known occurrence and can explain many “late cases” [30].

### 4.3. Ad Hoc Analysis

The observed increase in narcolepsy cases, as seen in our data, was greater than expected when considering the small number of patients receiving approved compensation due to Pandemrix-vaccination-related effects.

The number of cases that received financial compensation was only 450. Notably, The Medical Product Agency of Sweden (MPA) 2020 communicated a new evaluation of the number of Pandemrix-associated cases—a reduced number of 150–200 individuals. This was calculated by subtracting the estimated natural occurrence from the reported cases, (n = 394 at the end of 2020), thereby adjusting for “natural incidence”. https://www.lakemedelsverket.se/sv/behandling-och-forskrivning/vaccin/risker-med-vaccin/svininfluensan-pandemrix-och-narkolepsi (accessed on 19 January 2024).

Considering this new evaluation from MPA, we made a crude calculation of the possible excess cases taken from our data, post hoc: Table 1 illustrates that more than 1000 new cases were registered between 2009 and 2014 (five years). With an estimated “natural” incidence of around 70/year as recalculated from data by Wijnans et al. [16], only 350 cases would be expected to occur. Subsequently, if deaths are not considered, a conservative estimation would indicate 600–700 excess cases in our data from this period. Thus, even if those 450 patients who have received compensation for vaccination side effects are subtracted, hundreds of excess cases remain.

A potential explanation may be that the H1N1 influenza also increased the risk of narcolepsy because of changes to the immune system, as demonstrated by the increased morbidity of narcolepsy during the H1N1 pandemic in China affecting people who did not receive the Pandemrix vaccination [36]. The co-occurrence of subclinical infection and vaccination is also of interest in this respect. Such a phenomenon has been mentioned as a possible cause of the suspected geographic gradient of post-Pandemrix narcolepsy in Sweden: the vaccinations started from the north, and the infection entered the country from the south [6]. This resulted in early infections and late vaccinations in the southern part of the country, where the majority of vaccinated cases seemed to be located. There may also have been a previous under-diagnosing of narcolepsy, especially among children and teenagers. Narcolepsy among children was very rare before 2009, and poor knowledge may have diminished the chance of a correct diagnosis. The increased attention given to narcolepsy supported by the media may have finally increased the awareness of narcolepsy among the public, leading to more healthcare contacts in the affected group and introducing a bias into the study. However, the fact that children and teenagers mainly constituted the increased prevalence contradicts that overdiagnosis of narcolepsy could be a major cause. Such a bias would most probably affect all age groups similarly.

The magnitude of excess narcolepsy cases in Sweden and Finland after the pandemic is probably related to the extreme frequency of vaccination in the population, a true mass vaccination situation. The vulnerability allele HLA DQB1*0602, present in almost all vaccine-related cases, is to our knowledge not especially common in our country: The frequency of HLA DR15 was estimated to be 30% in Swedish general population controls [37].

Finally, the sex ratio deserves attention; for example, concerning the rapidly increasing quotient of women in the sex ratio of multiple sclerosis, another autoimmune disorder [38]. Environmental factors such as obesity, smoking, and shortage of vitamin D have been mentioned as possible causes [39]. In narcolepsy, on the other hand, we cannot yet state a similar sex-related development until a systematic evaluation of sex differences is performed in the future.

It is important to note the limitations in the present study, there is no information on the vaccination rate among narcolepsy cases, so the number of patients who did not receive the Pandemrix vaccination but who were still diagnosed with narcolepsy is unknown. Additionally, there is no information on concurrent swine flu in our material.

## 5. Conclusions

Our retrospective analysis of the data extracted from the NPR register in 2019 confirms the increase in narcolepsy. Since the Pandemrix vaccination status of the patients is not known, we could only crudely estimate the impact of the vaccination. Regardless, the apparent escalation seems to be greater than the 450 cases approved for compensation due to the onset of narcolepsy within two years. This represents a basis for looking for other reasons; for example, a prolonged latency before clinical onset or narcolepsy cases initiated by the H1N1 virus itself, through overt or subclinical infection. In addition, the impact of the infection and vaccination in combination should be considered. The prominent increase in children, adolescents, and young adults among the cases after 2009/2010, as demonstrated in our data, fits well with impressions of the recent situation in the narcolepsy polyclinic, where young patients tend to dominate in terms of frequency and also often the severity. This is of great concern, since an early onset of such a disease, affecting energy, concentration ability, and cognition, may be an obstacle to future personal and social development and consequently requires corresponding efforts from social and health care authorities.

## Figures and Tables

**Table 1:** The annual number of narcolepsy patients, prevalence, and sex ratio from the year 2005 to 2017 in the Swedish population.

	2005	2006	2007	2008	2009	2010	2011	2012	2013	2014	2015	2016	2017
Women													
Cases (nr.)	375	349	361	408	432	487	763	852	949	1042	1104	1136	1169
Inhabitants	4,561,202	4,589,734	4,619,006	4,652,637	4,691,668	4,725,326	4,756,021	4,789,988	4,830,507	4,875,115	4,920,051	4,981,806	5,037,580
Prevalence /100,000	8.22	7.60	7.82	8.77	9.21	10.31	16.04	17.79	19.65	21.37	22.44	22.80	23.21
Men													
Cases (nr.)	262	225	259	266	271	354	578	664	717	819	818	843	875
Inhabitants	4,486,550	4,523,523	4,563,921	4,603,710	4,649,014	4,690,244	4,726,834	4,765,905	4,814,357	4,872,240	4,930,966	5,013,347	5,082,662
Prevalence /100,000	5.84	4.97	5.67	5.78	5.83	7.55	12.23	13.93	14.89	16.81	16.59	16.82	17.22
All													
Cases (nr)	637	574	620	674	703	841	1341	1516	1666	1861	1922	1979	2044
Inhabitants	9,047,752	9,113,257	9,182,927	9,256,347	9,340,682	9,415,570	9,482,855	9,555,893	9,644,864	9,747,355	9,851,017	9,995,153	10,120,242
Prevalence /100,000	7.04	6.30	6.75	7.28	7.53	8.93	14.14	15.86	17.27	19.09	19.51	19.80	20.20
Sex ratio Women/Men	1.4	1.5	1.4	1.5	1.6	1.4	1.3	1.3	1.3	1.3	1.4	1.4	1.3

## Data Availability

Data supporting the reported results are available upon reasonable request from the first authors.

## Supplementary Materials

The following supporting information can be downloaded at: https://www.mdpi.com/article/10.3390/jcm13030652/s1, Table S1. Annual number of narcolepsy patients, population, and prevalence, divided into five age groups during the years 2005 to 2017 in the Swedish population.
