# Supplementary material for: Characterization of the Increase in Narcolepsy following the 2009 H1N1 Pandemic in Sweden
Source: J Clin Med. 2024 Jan 23;13(3):652. doi: 10.3390/jcm13030652 (PMC10856509; doi:10.3390/jcm13030652)
Supplement: Supplementary file 1 [file jcm-13-00652-s001.zip › jcm-2804176-supplementary.pdf]

Journal of
Clinical Medicine

MDPI

Supplementary Material: Characterization of the Increase in Narcolepsy following the 2009 H1N1 Pandemic in Sweden

Helena Gauffin, Inger Boström, Shala G. Berntsson, Anna Kristoffersson, Mats Fredriksson and Anne-Marie Landtblom

Table S1. Annual number of narcolepsy patients, population, and prevalence, divided into five age groups during the years 2005 to 2017 in the Swedish population.

Age		2005	2006	2007	2008	2009	2010	2011	2012	2013	2014	2015	2016	2017
0–19	Cases	46	46	46	64	62	182	440	551	580	618	587	517	477
	Population	2,159,156	2,169,309	2,178,761	2,183,810	2,187,975	2,183,564	2,176,646	2,176,486	2,189,728	2,209,269	2,239,615	2,290,606	2,339,515
	Prevalence *	2.1	2.1	2.1	2.9	2.8	8.3	20.2	25.3	26.5	28.0	26.2	22.6	20.4
20–39	Cases	200	165	196	194	238	248	380	412	496	596	685	737	845
	Population	2,319,620	2,322,803	2,331,671	2,355,172	2,391,779	2,429,690	2,457,057	2,484,477	2,512,096	2,541,030	2,566,502	2,610,359	2,641,608
	Prevalence *	8.6	7.1	8.4	8.2	10.0	10.2	15.5	16.6	19.7	23.5	26.7	28.2	32.0
40–59	Cases	174	139	132	164	168	160	211	240	257	307	299	368	364
	Population	2,430,317	2,434,859	2,441,379	2,442,425	2,444,696	2,450,925	2,467,063	2,483,080	2,498,938	2,522,210	2,539,026	2,554,909	2,568,266
	Prevalence *	7.2	5.7	5.4	6.7	6.9	6.5	8.6	9.7	10.3	12.2	11.8	14.4	14.2
60–79	Cases	200	193	218	219	209	219	263	273	279	288	292	278	285
	Population	1,651,496	1,696,032	1,740,154	1,781,827	1,821,847	1,854,487	1,883,871	1,913,702	1,946,385	1,975,438	2,004,224	2,032,385	2,058,183
	Prevalence *	12.1	11.4	12.5	12.3	11.5	11.8	14.0	14.3	14.3	14.6	14.6	13.7	13.8
≥ 80	Cases	17	31	28	33	26	32	47	40	52	52	59	79	73
	Population	487,163	490,254	490,962	493,113	494,385	496,904	498,218	498,148	497,717	499,408	501,650	506,894	512,670
	Prevalence *	3.5	6.3	5.7	6.7	5.3	6.4	9.4	8.0	10.4	10.4	11.8	15.6	14.2

* number per 100,000 inhabitants.
